# TMEDRP: decoding tumor-intrinsic and microenvironmental signatures for clinical drug response prediction

**DOI:** 10.1093/bioinformatics/btag680

**Published:** 2026-09-12

**Authors:** Yabin Kuang, Haochen Zhao, Yi Luo, Teng Sun, Hongdong Li, Guihua Duan, Jianxin Wang

**Affiliations:** School of Computer Science and Engineering, Central South University, Changsha, 410083, China; Hunan Provincial Key Lab on Bioinformatics, Central South University, Changsha, 410083, China; School of Computer Science and Engineering, Central South University, Changsha, 410083, China; Hunan Provincial Key Lab on Bioinformatics, Central South University, Changsha, 410083, China; School of Computer Science and Engineering, Central South University, Changsha, 410083, China; Hunan Provincial Key Lab on Bioinformatics, Central South University, Changsha, 410083, China; School of Computer Science and Engineering, Central South University, Changsha, 410083, China; Hunan Provincial Key Lab on Bioinformatics, Central South University, Changsha, 410083, China; School of Computer Science and Engineering, Central South University, Changsha, 410083, China; Hunan Provincial Key Lab on Bioinformatics, Central South University, Changsha, 410083, China; School of Computer Science and Engineering, Central South University, Changsha, 410083, China; Hunan Provincial Key Lab on Bioinformatics, Central South University, Changsha, 410083, China; School of Computer Science and Engineering, Central South University, Changsha, 410083, China; Hunan Provincial Key Lab on Bioinformatics, Central South University, Changsha, 410083, China

## Abstract

**Motivation:**

Clinical drug response prediction is constrained by the paucity of patient data, forcing a reliance on *in vitro* cell line models. Current transfer learning-based methods typically focus on learning domain-invariant representations, but often overlook the critical role of tumor microenvironment (TME) during cross-domain translation. Given that TME discrepancies between *in vitro* and *in vivo* settings are critical factors of therapeutic resistance, explicitly modeling the TME is essential to bridge the gap between preclinical models and patient-specific responses.

**Results:**

We propose TMEDRP, a novel two-stage learning framework that integrates tumor-intrinsic signatures and TME-specific components for enhanced clinical drug response prediction. TMEDRP introduces TME influences via a pathway-informed disentanglement strategy, a neural co-expression module, and an uncertainty-driven adaptation mechanism. Extensive benchmarks demonstrate that TMEDRP outperforms existing state-of-the-art methods. Importantly, it exhibits promising zero-shot generalization across unseen cancer lineages and novel compounds, suggesting its robustness across diverse scenarios. Furthermore, quantitative analysis of the model’s latent embeddings uncovers key tumor-intrinsic pathways and extrinsic TME factors that distinguish drug-sensitive from resistant cohorts. The identified shared and drug-specific molecular markers align with established clinical mechanisms, supporting the model’s biological interpretability and clinical utility.

**Availability and implementation:**

The code and results are available at https://github.com/kybinn/TMEDRP.

## 1 Introduction

The prediction of personalized drug response is a fundamental objective of precision oncology (Zhang *et al.* 2025). By leveraging patient molecular profiles, particularly transcriptomics, researchers endeavor to identify sensitivity to specific therapeutic compounds, thereby facilitating tailored treatment regimens that optimize survival outcomes and alleviate healthcare burdens (Wang *et al.* 2025, Partin *et al.* 2026). In recent years, large-scale preclinical screening initiatives, such as the Genomics of Drug Sensitivity in Cancer (GDSC) and the Cancer Cell Line Encyclopedia (CCLE), have comprehensively characterized the pharmacogenomic landscapes of thousands of cancer cell lines. The availability of these high-fidelity labeled datasets has catalyzed the development of computational architectures, exemplified by BinaryET (Branson *et al.* 2025), GraphCDR (Liu *et al.* 2022), and MSDRP (Zhao *et al.* 2023), which have achieved robust predictive performance across *in vitro* scenarios. Beyond transcriptome-based approaches, mutation-based frameworks have also been explored for drug response prediction. For example, ResGitDR (Ren *et al.* 2024) infers tumor-specific cellular signaling states from driver somatic genome alterations (SGAs) and uses these states for drug response prediction, providing a genome-informed view of therapeutic response.

However, these models often suffer from a decline in accuracy when translated to real-world patient cohorts. This performance degradation arises from a pronounced Out-of-Distribution (OOD) discrepancy between the source domain (cell lines) and the target domain (patient tumors) (Shi *et al.* 2025). Fundamentally, tumor proliferation dynamics diverge between synthetic culture media and human physiological environments, as *in vitro* cell lines fail to replicate the complex tumor microenvironment (TME), systemic immune interactions, and intricate vasculature present *in vivo* (Jo *et al.* 2018). Moreover, clinical samples are characterized by high biological heterogeneity and technical confounders, such as batch effects. Given that labeled clinical drug response data are scarce and fragmented, direct large-scale supervised training on patient data remains infeasible, thereby limiting the generalizability of existing computational frameworks.

To bridge this preclinical-to-clinical gap, methodologies based on unsupervised domain adaptation (UDA) provide a robust framework by extracting domain-invariant representations without requiring target-domain labels. Early efforts primarily relied on statistical alignment, utilizing techniques like ComBat (Geeleher *et al.* 2014) for batch correction or PCA-based frameworks including PRECISE (Mourragui *et al.* 2019) and TRANSACT (Mourragui *et al.* 2021), to align latent subspaces and minimize geometric distribution divergences.

Driven by advancements in deep learning, adversarial learning has been widely integrated into drug response modeling to enhance predictive robustness. Velodrome (Sharifi-Noghabi *et al.* 2021) proposed a OOD generalization framework that balances domain-invariance and hypothesis-invariance by combining consistency and alignment losses. To address vanishing gradient issues typical of adversarial training, PANCDR (Kim *et al.* 2024) adopted a “two-step” optimization strategy in lieu of the standard gradient reversal layer, thereby enhancing generalization in multi-drug scenarios. Furthermore, TRANSPIRE-DRP (Yang *et al.* 2025) demonstrated that large-scale unlabeled pre-training, coupled with adversarial learning, can effectively facilitate knowledge transfer from Patient-Derived Xenograft (PDX) models to clinical cohorts. More recently, research focus has shifted toward refined feature disentanglement and clinical knowledge integration. CODE-AE (He *et al.* 2022) leverages self-supervised learning to decouple drug-sensitive biomarkers from domain-specific signals, while DAPL (Peng *et al.* 2025) incorporates prototypical learning and orthogonal constraints to improve performance. Similarly, TransDRP (Liu and Li 2025) presents a knowledge-guided architecture that encodes molecular similarity via multi-label graph neural networks and employs a global-local adversarial strategy to strengthen alignment within similar cancer types. MACB-DRP (Bai *et al.* 2026) introduced a hierarchical cross-domain adaptation framework that coordinates distribution alignment at the tissue, drug, and sample levels, while employing contrastive domain isolation and bilinear fusion to preserve biological differences and capture gene-drug interactions.

Despite these advancements, a critical bottleneck remains: most existing methodologies treat the systemic discrepancies between *in vitro* and *in vivo* environments as stochastic noise rather than explicitly modeling the underlying biological mechanisms. Drug2TME (Zhai and Liu 2024) made an initial attempt to incorporate the tumor microenvironment concept by using domain separation networks to deconstruct tumor features. DiSyn (Li *et al.* 2025) further treated these components as drug-unspecific patient features, leveraging them to generate synthetic samples to address data scarcity. However, these models typically attribute cross-domain discrepancies to latent TME factors, which may lack direct biological interpretability. Moreover, effectively isolating and harnessing TME-specific features within the constraints of a cross-domain context remains a challenge.

This paper introduces **T**umor **M**icroenvironment-aware **D**rug **R**esponse **P**rediction (TMEDRP), a pathway-informed transfer learning framework that explicitly models the TME to enhance precision therapy. Specifically, leveraging Gene Set Enrichment Analysis (GSEA) to identify tumor-intrinsic and TME-related gene sets, the framework employs a Transformer-based pathway encoder for feature extraction. In the pre-training phase, TMEDRP achieves cross-domain alignment of tumor-intrinsic features via adversarial training while isolating TME components through orthogonal constraints. During the adaptation stage, a neural co-expression module is introduced to capture gene interactions, reintegrating decoupled features to mitigate biological information loss inherent in gene-level partitioning. To further decode the biological context of clinical data, we employ an uncertainty-driven adaptation strategy utilizing Monte Carlo Dropout and Max-Min Entropy optimization. This allows the model to distill robust TME representations from extensive unlabeled clinical cohorts. Extensive evaluations show that TMEDRP achieves competitive performance across multiple benchmarks, demonstrating its generalizability and interpretability.

## 2 Materials and methods

### 2.1 Datasets

To ensure methodological consistency and comparability with existing benchmarks, the data processing pipeline follows established protocols for cross-domain drug response prediction.

The pre-training phase incorporates transcriptomic profiles from 1517 cancer cell lines and 9808 patient tumor samples, sourced from the CCLE and TCGA repositories, respectively. In accordance with established cross-domain protocols, we adopted the preprocessed 1306-gene expression feature space following TransDRP (Liu and Li 2025). Enrichment analysis against the MSigDB ‘Hallmark 2020’ collection identified 20 significantly enriched TME-related pathways, yielding 574 unique TME-associated genes. Further screening against the MSigDB ‘Oncogenic Signatures’ collection retained 85 signatures encompassing 481 of these 574 genes. Detailed pathway annotations are provided in Supplementary Tables S10 and S11. Drug chemical structures were retrieved as SMILES strings from the PubChem database.

For the adaptation stage, $IC_{50}$ values were standardized into *z*-scores. Following standard practice, a threshold of 0 was employed to define therapeutic phenotypes, classifying negative *z*-scores as sensitive and non-negative values as resistant. The model’s clinical utility was validated using 1186 labeled patient records across nine drugs. Clinical response labels were derived from RECIST guidelines (Ding *et al.* 2016), where Complete Response and Partial Response were classified as sensitive, while Stable Disease and Progressive Disease were categorized as resistant.

Furthermore, the model’s robustness was assessed through two additional test scenarios. To evaluate cross-tissue generalizability, a clinical cohort from the Burkitt Lymphoma Genome Sequencing Project (CGCI-BLGSP) was retrieved from the GDC Data Portal as an independent validation set. Additionally, we collected response data for three novel drugs within the existing patient domain to evaluate the model’s extensibility to unseen chemical spaces. Comprehensive details regarding all datasets are provided in Supplementary Tables S1-S2 and Supplementary Fig. S1.

**Figure 1:**
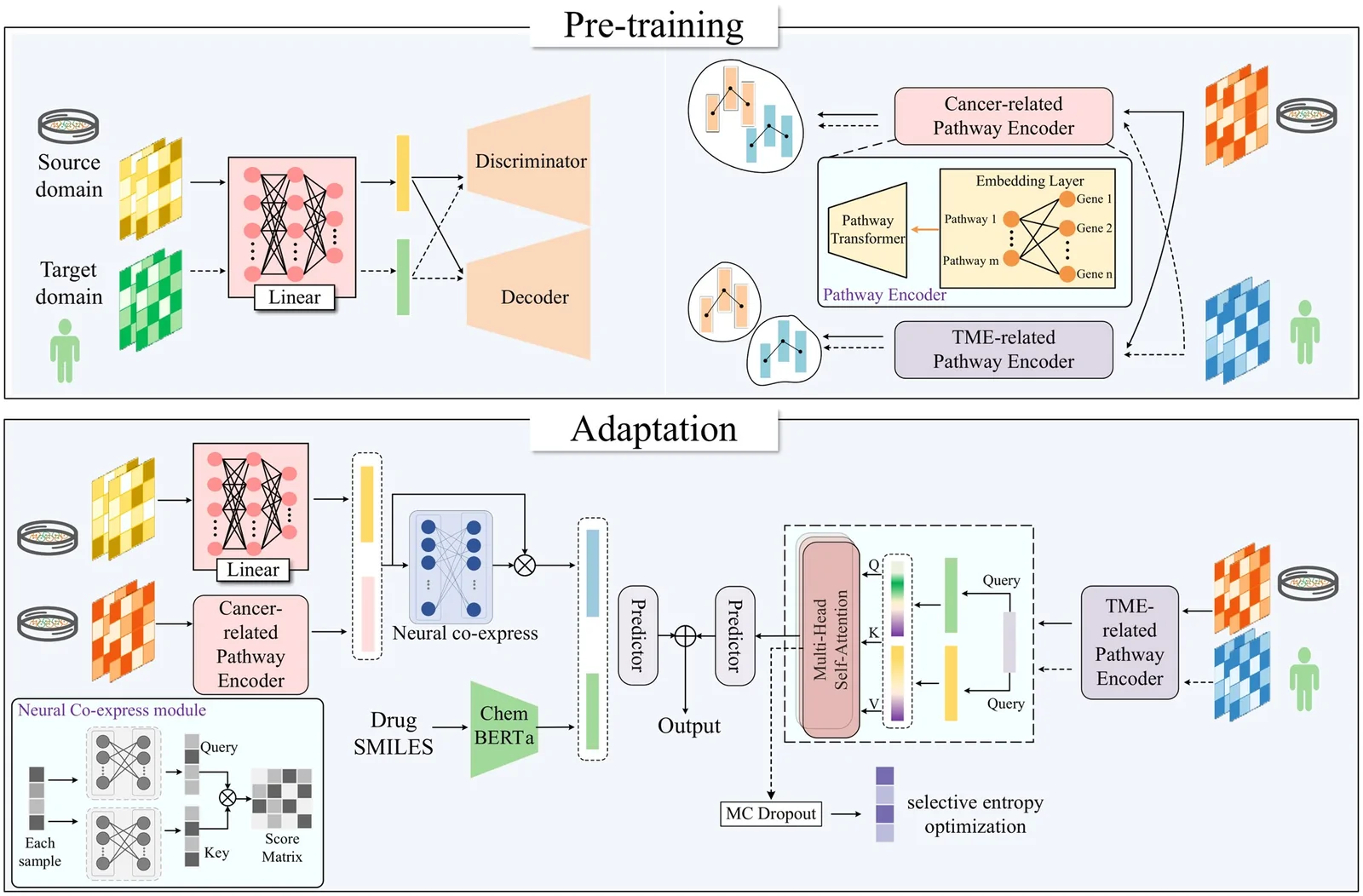
The framework of TMEDRP. (i) Pre-training phase. Guided by GSEA-derived biological priors, this phase decouples transcriptomic profiles into domain-invariant tumor-intrinsic signatures and domain-specific TME representations. Adversarial learning is employed to align shared oncogenic signals, while TME-specific features are extracted via a knowledge-guided pathway encoder. (ii) Adaptation phase. A neural co-expression module first recovers regulatory dependencies, followed by a dual-branch prediction head that combines tumor-intrinsic and TME-related influences. Besides, an uncertainty-driven strategy using Monte Carlo Dropout is further applied to distill robust biological signals from unlabeled clinical data.

### 2.2 Pre-training phase

As illustrated in Figure 1, the goal of pre-training phase is to preserve shared cancer-intrinsic feature while identifying TME-related discrepancies, effectively decoupling complex biological signals into distinct components.

#### 2.3 Cross-domain adversarial training

Latent representations from the source domain ($D_{s}$, cell lines) and target domain ($D_{t}$, patient tumors) are aligned via adversarial training (Ganin *et al.* 2016) to extract domain-invariant feature. A three-layer multilayer perceptron (MLP), denoted as $G_{f}$, serves as the feature extractor for tumor-intrinsic gene expression $X_{\mathrm{tum}}$. The extracted features are subsequently passed to a domain discriminator $G_{d}$ to identify the data origin. To facilitate adversarial learning, a gradient reversal layer (GRL) is integrated between $G_{f}$ and $G_{d}$, which reverses the gradient flow during backpropagation. This mechanism encourages the feature extractor to evolve in a direction that deceives the discriminator. The adversarial optimization objective employs a binary cross-entropy loss to minimize the domain discrepancy:


(1) $$\begin{array}{cc} L_{\mathrm{adv}}=-\sum_{X_{\mathrm{tum}}^{s}\in D_{s}}\;\text{log}\;G_{d}(E_{\mathrm{tum}}^{s})-\sum_{X_{\mathrm{tum}}^{t}\in D_{t}}\;\text{log}\;(1-G_{d}(E_{\mathrm{tum}}^{t})) & \\ & \text{with}\;E_{\mathrm{tum}}=G_{f}(X_{\mathrm{tum}}) \end{array}$$

where $X_{\mathrm{tum}}^{s}\in D_{s}$ and $X_{\mathrm{tum}}^{t}\in D_{t}$ denote the tumor-intrinsic expression profiles sampled from the source and target domain, respectively. Simultaneously, a decoder is introduced to restore the features to the original input space and the reconstruction loss is defined as follows:


(2) $$L_{\mathrm{rec}}=\frac{1}{n}\sum \|X_{\mathrm{tum}}-{\hat{X}}_{\mathrm{tum}}{\|}_{F}^{2}$$

#### 2.3.1 TME-related feature extracting

While core oncogenic signaling pathways are relatively conserved, TME components exhibit significant domain shifts between *in vitro* and *in vivo* settings, as illustrated in Fig. S2. Explicitly modeling the TME is essential for identifying domain-specific discrepancies that often confound drug response transition.

Traditional transcriptomics-based TME representation methods primarily rely on deconvolution algorithms to estimate cellular proportions. Notably, several advanced deconvolution frameworks leverages enrichment-based principles to capture biological signatures. Therefore, we utilize gene set enrichment analysis (GSEA) to map gene expression into TME-related functional pathways. A knowledge-guided pathway encoder is designed to transform gene expression into high-level functional entities. The encoder utilizes a biological prior mask matrix $M_{\mathrm{tme}}\in {\left\{0,1\right\}}^{N_{p}\times N_{g}}$ to constrain the feature projection, where $M_{ij}=1$ if gene *j* belongs to pathway *i*, and 0 otherwise. This ensures that each pathway aggregates information exclusively from its constituent genes, yielding biologically meaningful features. The transformation from gene-level expression $X_{\mathrm{tme}}$ to the final microenvironment representation $Z_{\mathrm{tme}}$ is formulated as:


(3) $$\begin{array}{cc} Z_{\mathrm{tme}}=\text{Pooling}\left(\text{Transformer}\left(H_{\mathrm{tme}}\right)\right) & \\ & \text{with}\;H_{\mathrm{tme}}=\text{LayerNorm}\left(F(X_{\mathrm{tme}},M_{\mathrm{tme}})\right) \end{array}$$

where F(·) denotes the constrained linear projection defined by the mask $M_{\mathrm{tme}}$, subsequently normalized via a LayerNorm layer to generate the initial pathway embeddings $H_{\mathrm{tme}}$. A transformer encoder is introduced to capture 64-dimensional inter-pathway dependencies $Z_{\mathrm{tme}}$. Specifically, the pathway encoder employs a mask-constrained embedding layer followed by a two-layer Transformer with two attention heads. Then we minimize the cross-domain orthogonal loss to project the TME features into disparate latent spaces:


(4) $$L_{\mathrm{ortho}}=|Z_{\mathrm{tme}}^{s}\cdot {\left(Z_{\mathrm{tme}}^{t}\right)}^{⊤}{|}^{2}$$

where $Z_{\mathrm{tme}}^{s}$ and $Z_{\mathrm{tme}}^{t}$ are TME-related representations from the source and target domains, respectively.

It is important to recognize that genes involved in TME-related pathways often exhibit functional pleiotropy, playing dual roles in both external regulation and intrinsic tumor processes (e.g. metabolic reprogramming). Therefore, we employ a parallel pathway encoder to preserve critical oncogenic signals during the TME-specific decomposition phase. By utilizing an additional oncogenic mask matrix $M_{\mathrm{tum}}$ derived from the MSigDB Oncogenic Signatures, the shared tumor-intrinsic pathway-level representations $Z_{\mathrm{tum}}$ are captured as follows:


(5) $$\begin{array}{cc} Z_{\mathrm{tum}}=\text{Pooling}\left(\text{Transformer}\left(H_{\mathrm{tum}}\right)\right) & \\ & \text{with}\;H_{\mathrm{tum}}=\text{LayerNorm}\left(F(X_{\mathrm{tme}},M_{\mathrm{tum}})\right) \end{array}$$

Following this parallel enrichment, the CORAL loss is applied to align oncogenic features $Z_{\mathrm{tum}}$ across domains:


(6) $$L_{\text{coral}}={\left\|C_{S}-C_{T}\right\|}_{F}^{2},$$

where $C_{S}$ and $C_{T}$ denote the covariance matrices of the oncogenic representations $Z_{\mathrm{tum}}$ from the source and target domains, respectively. This parallel enrichment ensures that information pertinent to tumor proliferation is retained in the cancer-intrinsic latent space, even as microenvironment-related variations are identified and isolated.

The total optimization objective $L_{\mathrm{pre}}$ for the pre-training phase is defined as:


(7) $$L_{\mathrm{pre}}=L_{\mathrm{adv}}+L_{\mathrm{rec}}+L_{\mathrm{coral}}+\alpha L_{\mathrm{ortho}}$$

where α is set to 0.1 to balance the numerical scale of $L_{\mathrm{ortho}}$ with the other loss components.

### 2.4 Adaptation phase

This phase integrates the pre-trained disentangled embeddings into a unsupervised domain adaptation framework for clinical drug response prediction.

#### 2.4.1 Cancer-intrinsic sensitivity

In this branch, we leverage two complementary representations of the tumor’s internal state derived from the pre-training stage: $E_{\mathrm{tum}}$, the 64-dimensional domain-invariant latent embedding extracted directly from raw gene expressions $X_{\mathrm{tum}}$ via adversarial alignment; and $Z_{\mathrm{tum}}$, the shared functional embedding derived from $X_{\mathrm{tme}}$ via oncogenic pathway enrichment.

While these aligned features provide a robust foundation, simply concatenating them may overlook the complex functional dependencies and regulatory associations inherent to tumor biology. To address this, we propose a Neural Co-expression Module to reconstruct the latent associations between these decoupled representations, aiming to capture the comprehensive domain-invariant oncogenic signals. A dynamic co-expression matrix $A\in R^{128\times 128}$ for each individual sample was computed via a projected outer product:


(8) $$\begin{array}{c} A=\text{Softmax}\left(\phi ([E_{\mathrm{tum}},Z_{\mathrm{tum}}])\cdot \psi {\left([E_{\mathrm{tum}},Z_{\mathrm{tum}}]\right)}^{T}\right) \end{array}$$

where [·] denotes the concatenation operation, while ϕ and ψ represent learnable linear transformations that map the joint representation into Query and Key spaces, respectively. The softmax operation is applied row-wise, each entry $A_{ij}$ quantifies the latent dependency between the *i*-th and *j*-th feature components. The features are subsequently refined through a message-passing operation:


(9) $$H_{\mathrm{tum}}=A\cdot [E_{\mathrm{tum}},Z_{\mathrm{tum}}]$$

Through this operation, each feature is re-weighted based on the adjacency matrix *A* within the oncogenic network, alleviating information fragmentation caused by the initial partitioning of gene sets. Besides, to ensure the consistency of the relationship matrix *A* across domains, we minimize the squared Frobenius norm between the prototypical co-expression centroids of identical cancer types from CCLE and TCGA:


(10) $$L_{\mathrm{type}}=\sum_{k\in K}\|{\bar{A}}_{s}^{k}-{\bar{A}}_{t}^{k}{\|}_{F}^{2}$$

where ${\bar{A}}_{s}^{k}$ and ${\bar{A}}_{t}^{k}$ denote the mean co-expression matrices for cancer type *k* in the source and target domains, respectively, and K denotes the set of cancer types shared between the source and target domains.

To incorporate drug properties, the chemical semantics are captured via ChemBERTa (Chithrananda *et al.* 2020), a pre-trained chemical language transformer that yields high-dimensional molecular embeddings $Z_{\mathrm{drug}}$ from SMILES strings. $H_{\mathrm{tum}}$ is then concatenated with $Z_{\mathrm{drug}}$ to form a drug-disease joint embedding. Subsequently, a feed-forward neural network ${\text{FF}}_{1}(\cdot )$ is applied to generate a tumor-intrinsic sensitivity prediction:


(11) $${\hat{y}}_{\mathrm{tum}}={\text{FF}}_{1}([H_{\mathrm{tum}},Z_{\mathrm{drug}}])$$

#### 2.4.2 TME-mediated sensitivity

To incorporate the clinical impact of the tumor microenvironment, we employ a dual cross-attention mechanism, where the TME embedding $Z_{\mathrm{tme}}$ acts as the query, separately attending to the drug embedding $Z_{\mathrm{drug}}$ and the oncogenic embedding $E_{\mathrm{tum}}$. The resulting are fused via a self-attention layer and a feed-forward neural network ${\text{FF}}_{2}(\cdot )$ to yield the TME-mediated prediction ${\hat{y}}_{\mathrm{tme}}$.

A fundamental challenge arises from the fact that TME signals in cell lines are often sparse or fail to represent the complex clinical condition. To bridge this gap, we implement an uncertainty-driven strategy using unlabeled patient data from TCGA. For each clinical sample, two forward passes are executed under Monte Carlo Dropout perturbations, yielding an average probability $\bar{p}$. We partition samples into two mutually exclusive subsets: a consistent set C (where both perturbations yield the same classification decision) and an uncertain set U (where decisions diverge). A consistency-weighted entropy loss is defined as:


(12) $$\begin{array}{cc} L_{\mathrm{Ent}}=\frac{1}{\left|C\right|}\sum_{i\in C}H({\bar{p}}_{i})-\frac{1}{\left|U\right|}\sum_{j\in U}H({\bar{p}}_{j}) & \\ & \text{with}\;H(\bar{p})=-[\bar{p}\;\text{log}(\bar{p})+(1-\bar{p})\;\text{log}(1-\bar{p})] \end{array}$$

where H(·) denotes the binary entropy, and |·| represents the cardinality of the subset. This loss encourages the model to self-correct by identifying reliable clinical patterns: it minimizes entropy in C to reinforce robust TME-drug associations, while maximizing entropy in U to mitigate the impact of biased or noisy clinical patterns.

#### 2.4.3 Multi-head prediction

The final drug response prediction is formulated as a weighted ensemble of two branches:


(13) $$\hat{y}=\sigma ({\hat{y}}_{\mathrm{tum}}+{\hat{y}}_{\mathrm{tme}})$$

where σ(·) is the sigmoid function. The primary objective is to minimize the binary cross-entropy between the predictions and the ground-truth labels:


(14) $$L_{\mathrm{bce}}=-\frac{1}{N}\sum_{i=1}^{N}\left[y_{i}\;\text{log}({\hat{y}}_{i})+(1-y_{i})\;\text{log}(1-{\hat{y}}_{i})\right]$$

where *N* denotes the total number of samples.

Moreover, during the adaptation phase, the parameters of the pre-trained encoders are fine-tuned alongside the prediction heads to achieve task-specific alignment. The decoder and domain discriminator are removed at this stage. The global objective is defined as follows:


(15) $$L_{\mathrm{total}}=L_{\mathrm{bce}}+\underset{\text{TME}\;\text{Adaptation}}{\underset{︸}{\lambda_{1}L_{\mathrm{ortho}}+\lambda_{2}L_{\mathrm{Ent}}}}+\underset{\text{Oncogenic}\;\text{Alignment}}{\underset{︸}{\lambda_{3}L_{\mathrm{coral}}+\lambda_{4}L_{\mathrm{type}}}}$$

where $L_{\mathrm{ortho}}$ and $L_{\mathrm{Ent}}$ manage microenvironmental discrepancies, while $L_{\mathrm{coral}}$ and $L_{\mathrm{type}}$ facilitate the transfer of cancer-intrinsic signals. To balance the contributions of different loss components, the weighting factors $\lambda_{1},\lambda_{2}$, $\lambda_{3}$ and $\lambda_{4}$ were set to 0.01, 0.1, 10 and 1, respectively. To assess the sensitivity to these loss weights, we further performed a one-factor-at-a-time perturbation analysis, with the results provided in Fig. S3.

### 2.5 Experimental settings

Clinical prediction was formulated as transductive unsupervised domain adaptation (UDA), using labeled cell lines as the source domain and unlabeled patient profiles as the target domain. The area under the receiver operating characteristic curve (AUROC) and the area under the precision-recall curve (AUPR) serve as the primary performance indicators. Furthermore, the F1-score and accuracy (ACC) are included as additional metrics. Models were trained via five-fold cross-validation on cell lines, with early stopping guided by validation AUROC. Crucially, target clinical labels were strictly excluded from model training and hyperparameter tuning.

The baseline methods are categorized into single-drug and multi-drug frameworks. The single-drug models requiring separate models for each unique compound, include Velodrome (Sharifi-Noghabi *et al.* 2021), CODE-AE (He *et al.* 2022), WISER (Shubham *et al.* 2024), and TRANSPIRE-DRP (Yang *et al.* 2025). In contrast, multi-drug models incorporate chemical structure information to make response predictions across diverse drug scenarios, including drug2TME (Zhai and Liu 2024), PANCDR (Kim *et al.* 2024), DAPL (Peng *et al.* 2025), TransDRP (Liu and Li 2025), MACB-DRP (Bai *et al.* 2026), and our proposed TMEDRP. These state-of-the-art methods were selected due to their demonstrated capacity to address domain discrepancies in drug response prediction. For a fair comparison, all methods were implemented using the optimized hyperparameters specified in their original references. Predicted probabilities were binarized using a fixed threshold of 0.5 across all drugs and models, without optimization against clinical labels.

## 3 Results

### 3.1 Comparison with other methods

**Figure 2:**
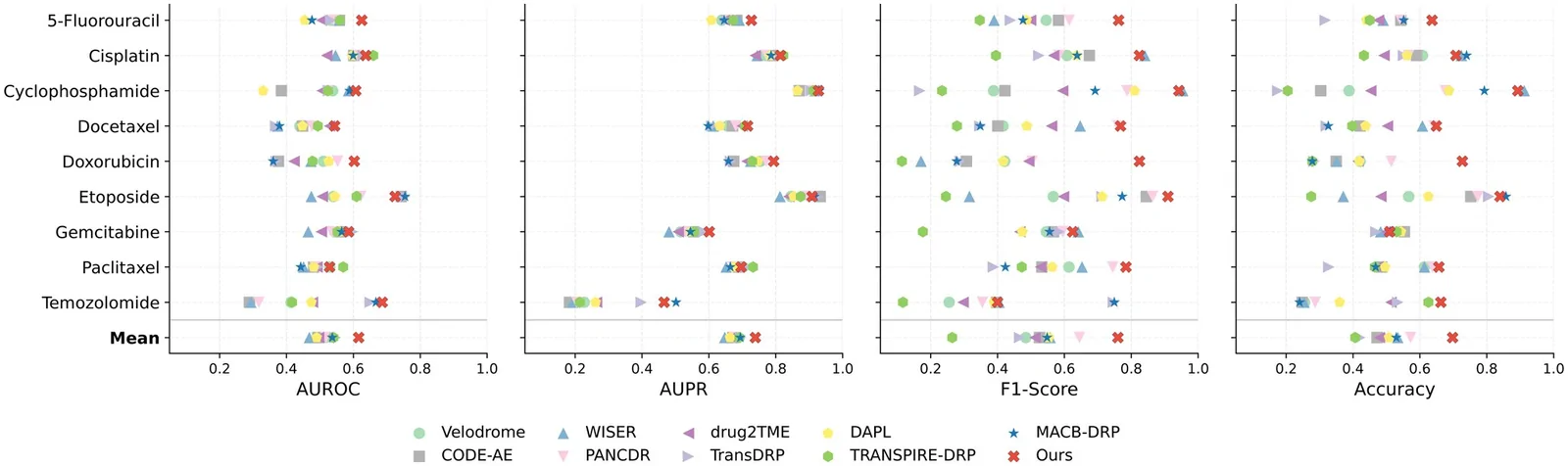
Performance comparison of TMEDRP and baseline methods across nine therapeutic agents.

Figure 2 and Table 1 present the prediction performance of the ten evaluated methods across nine therapeutic agents. Overall, TMEDRP achieves the most robust performance, outperforming both single-drug and multi-drug baselines across the four evaluation metrics: AUROC, AUPR, F1-score, and ACC. Specifically, our proposed framework achieves the highest AUROC and AUPR scores on the majority of the tested drugs, including Doxorubicin, Docetaxel, and Temozolomide. Detailed performance results for all approaches are documented in Supplementary Tables S3–S6. To assess performance consistency across therapeutic agents, we performed two-sided Wilcoxon signed-rank tests across all nine drugs, comparing TMEDRP against the strongest baseline for each metric (TransDRP for AUROC and AUPR; PANCDR for F1 and ACC). TMEDRP achieved statistically significant improvements across all metrics, with *P* values of .0352, .0391, .0039, and .0078 for AUROC, AUPR, F1-score, and ACC, respectively. Fold-wise metrics are provided in Fig. S4.

**Table 1:** Performance comparison in terms of the average AUROC, AUPR, F1, and ACC across nine therapeutic agents.

Model	**AUROC** ↑	**AUPR** ↑	**F1** ↑	**ACC** ↑
Velodrome	0.517	0.670	0.484	0.484
CODE-AE	0.494	0.672	0.525	0.472
WISER	0.506	0.679	0.509	0.543
PANCDR	0.518	0.676	0.645	0.572
drug2TME	0.496	0.667	0.512	0.479
TransDRP	0.546	0.695	0.364	0.466
DAPL	0.490	0.663	0.553	0.508
TRANSPIRE	0.540	0.691	0.265	0.407
MACB-DRP	0.537	0.694	0.529	0.548
**Ours**	**0.616**	**0.739**	**0.760**	**0.698**
Wilcoxon *P*-value	.0352	.0391	.0039	.0078

Bold indicates the best performance, while underline indicates a suboptimal performance. Statistical significance was assessed using two-sided paired Wilcoxon signed-rank tests across the nine therapeutic agents against the strongest baseline for each metric.

**Figure 3:**
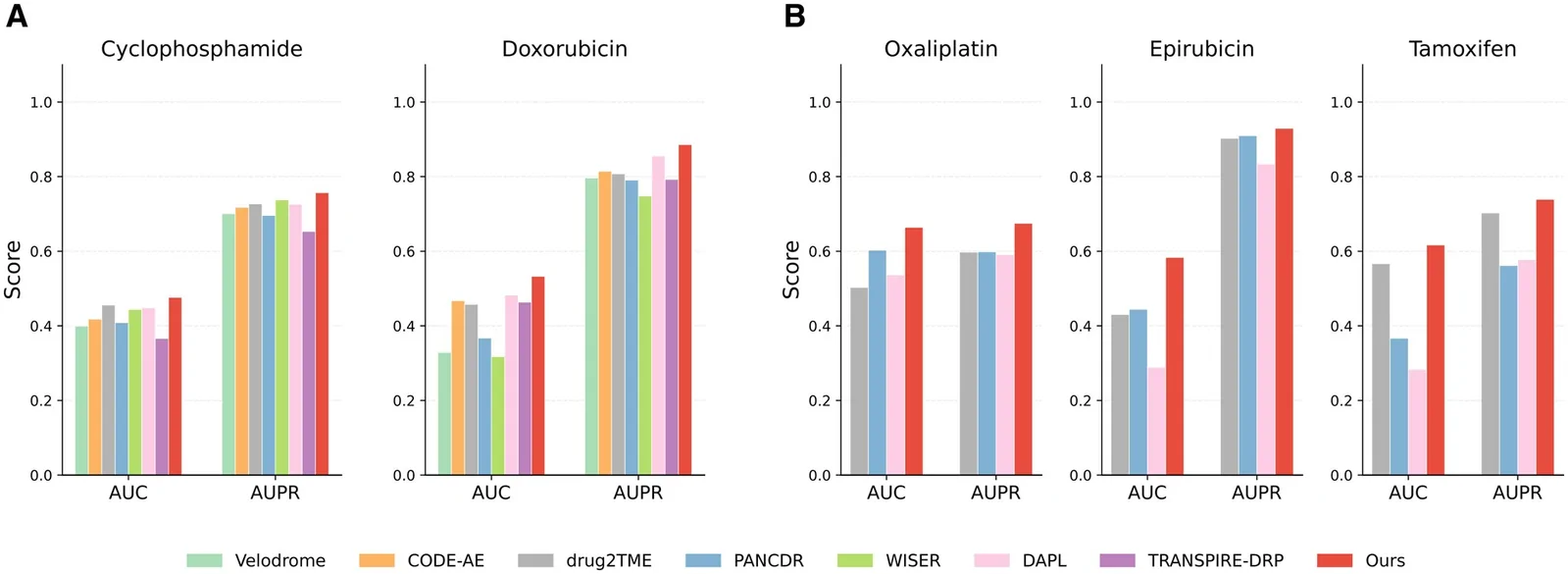
(A) External validation on independent clinical cohorts of B-cell lymphoma. (B) Zero-shot generalization to unseen chemical compounds.

To evaluate the model’s capacity for cross cancer type generalization, we conducted external validation using independent clinical cohorts of B-cell lymphoma retrieved from the CGCI. This task evaluates zero-shot generalization to the unseen B-cell lymphoma lineage using Cyclophosphamide and Doxorubicin. All models retained their original weights and decision boundaries without any target-domain fine-tuning. Performance was evaluated using threshold-independent metrics, namely AUROC and AUPR, to ensure a reliable assessment in this cross-dataset context. As illustrated in Fig. 3A, TMEDRP demonstrates better performance across both drugs. Notably, TransDRP and MACB-DRP were excluded because their fixed-drug, multi-label architectures lack the structural flexibility required for independent evaluation on these tasks. Detailed results are documented in Table S7.

To evaluate the model’s ability to generalize to unseen drugs, we tested TMEDRP on three compounds entirely excluded from the training set: Oxaliplatin, Epirubicin, and Tamoxifen.The comparison is restricted to multi-drug baselines (drug2TME, PANCDR, and DAPL), as single-drug models are limited to their training drug sets. As shown in Fig. 3B and Table S8, TMEDRP outperforms all multi-drug baselines on Oxaliplatin, Epirubicin, and Tamoxifen in both AUROC and AUPR. This supports its capacity to generalize to new medication scenarios and highlights its potential for identifying novel therapeutic candidates.

### 3.2 Ablation study

To evaluate the contribution of each module, we compare the full TMEDRP with five architectural variants:

- **Variant 1** performs global cross-domain adversarial training on all genes without explicit TME decomposition or TME-related prediction.
- **Variant 2** replaces the pathway-informed mask constraint with a linear projection incorporating orthogonal constraints.
- **Variant 3** integrates the pathway-informed TME decomposition, while removing the parallel cancer-related pathway encoder.
- **Variant 4** removes the proposed Neural Co-expression Module, evaluating the impact of latent feature associations.
- **Variant 5** disables the uncertainty-driven adaptation on unlabeled TCGA samples.

**Figure 4:**
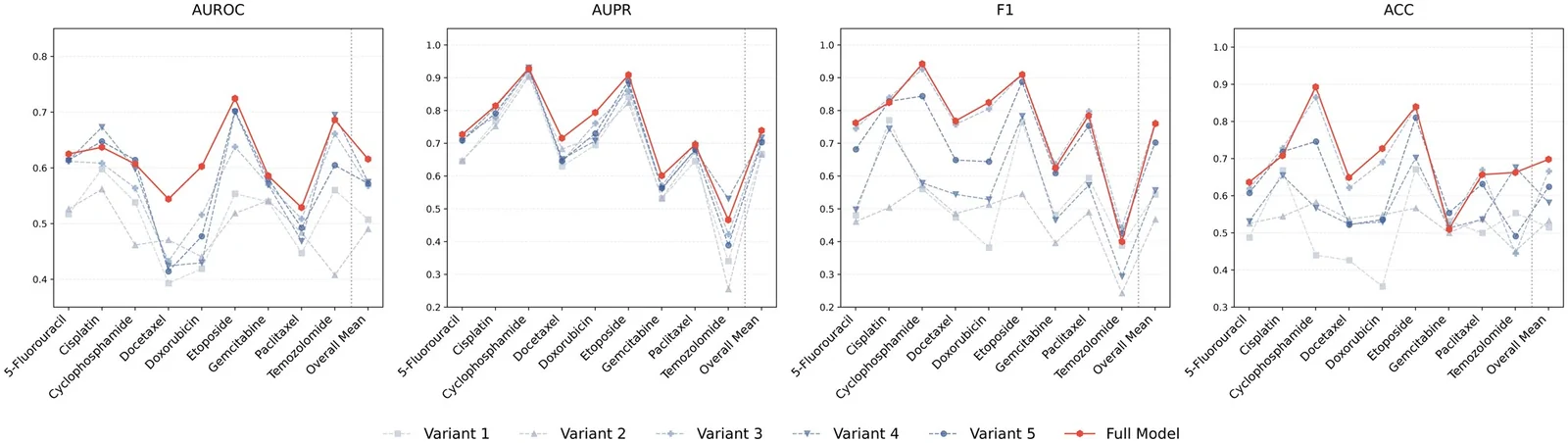
Ablation study of TMEDRP architectural components.

Figure 4 and Table S9 demonstrate that the full TMEDRP model achieves better performance over all variants across the nine tested drugs, highlighting the synergistic effect of the proposed modules. Specifically, the significant performance gap between the full model and **Variant 1** (which lacks TME decomposition) highlights that explicitly decoupling the tumor microenvironment is far more effective than relying on global gene expression alone. The lower scores of **Variant 2** and **Variant 3** indicate that pathway-informed priors are vital for capturing complex tumor-environment interactions, thereby demonstrating its superiority over the empirical orthogonal mapping strategies adopted in drug2TME. **Variant 4** exhibits a marked decline in F1-score and ACC, particularly for drugs like Docetaxel and Doxorubicin. This suggests that simulating gene co-expression interactions is crucial for addressing information lost during feature partitioning. Unsupervised clustering analysis (Fig. S5) further demonstrated that the proposed module significantly improves the compactness and separability of patient embeddings, facilitating more effective capture of high-order transcriptional features. Moreover, the performance decline in **Variant 5** proves that distilling knowledge from unlabeled clinical cohorts via uncertainty-driven optimization effectively enhances the model’s generalization to real-world patient data.

Overall, the ablation study confirms that the integration of pathway-informed priors, TME decoupling, and clinical adaptation is essential for bridging the preclinical-to-clinical gap in drug response prediction.

### 3.3 Case study

#### 3.3.1 Interpretability of TME representations

To verify the biological relevance of the learned TME embeddings, we selected Breast Cancer (BRCA) as a representative case study due to its substantial sample size. We performed a Pearson correlation analysis between the model’s encoded TME representations (compressed via the first principal component) and established microenvironment scoring systems (CIBERSORT and ESTIMATE). Across the five evaluated drug-specific TME representations, PC1 explained 86.54%–91.69% of the total embedding variance, indicating that it captured the dominant variation in the learned TME latent space. As shown in Fig. S6, the TME embeddings exhibit significant concordance with key stromal and immune features across different drug-response groups. Specifically, a consistent and significant correlation with the StromalScore was observed across all treatment cohorts. Furthermore, drug-specific patterns were observed in the Cyclophosphamide group, characterized by a uniquely significant correlation between the TME embedding and Regulatory T cells (Tregs). Given that clinical evidence indicates low-dose Cyclophosphamide can selectively deplete Tregs to counteract immunosuppression (Ahlmann and Hempel 2016), this observation suggests that TMEDRP potentially captures the immunomodulatory effects of Cyclophosphamide within its latent space, aligning with its known clinical mechanism.

#### 3.3.2 Molecular markers of predicted sensitivity and resistance

**Figure 5:**
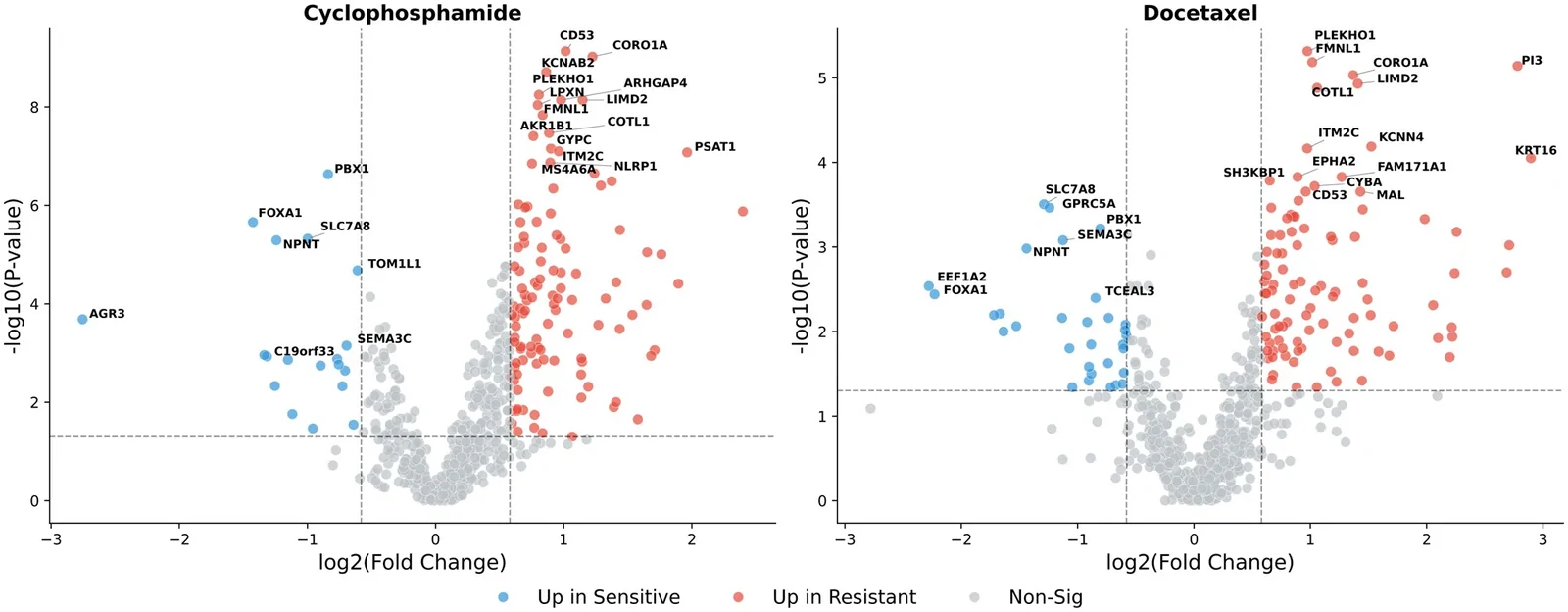
Differential gene expression analysis between predicted sensitive and resistant cohorts. Volcano plots show the DEGs for Cyclophosphamide (left) and Docetaxel (right). Red and blue dots represent significantly up-regulated genes in the resistant and sensitive groups, respectively ($P<0.05,|{\;\text{log}\;}_{2}\text{FC}|>0.58$). Grey dots denote non-significant genes.

To investigate the biological mechanisms of TMEDRP’s predictions, we identified differentially expressed genes (DEGs) between patient cohorts predicted as sensitive and resistant to Cyclophosphamide and Docetaxel. As illustrated in Fig. 5, consistent molecular signatures emerged across both drug treatments. Specifically, the sensitivity-associated groups were characterized by significant up-regulation of ‘FOXA1’ and ‘SLC7A8’. ‘FOXA1’ serves as a hallmark of well-differentiated Luminal-type breast cancer (Badve *et al.* 2007), while ‘SLC7A8’ is recognized as a favorable prognostic indicator in ER+ subtypes (El Ansari *et al.* 2020). The enrichment of these markers suggests that TMEDRP identifies metabolically active, Luminal-like malignant cells as being more susceptible to conventional chemotherapy. In contrast, resistant cohorts up-regulated ‘CORO1A’ and ‘LIMD2’ to drive cytoskeletal remodeling and EMT, enhancing cell plasticity and motility to evade drug-induced apoptosis (Wang *et al.* 2025).

Beyond these shared features, the model captured drug-specific resistance mechanisms. For instance, the Cyclophosphamide-resistant group demonstrated a distinct elevation of ‘PSAT1’, reflecting a metabolic adaptation where enhanced serine biosynthesis provides necessary precursors for DNA damage repair (Montrose *et al.* 2021). Conversely, Docetaxel resistance was marked by the upregulation of ‘FAM171A1’, which has been identified as a potential oncogene that influences actin cytoskeletal organization (Soylemez and Ayan 2025). Since Docetaxel acts by binding tubulin, the signaling crosstalk between actin and microtubules may facilitate a structural bypass that allows cells to evade drug-induced mitotic arrest (Dogterom and Koenderink 2019).

#### 3.3.3 Pathway signatures of predicted sensitivity and resistance

We further evaluated TMEDRP’s clinical utility on an unlabeled cohort of 856 BRCA patients from TCGA. By ranking patients based on their predicted response scores for Cyclophosphamide, we categorized the top and bottom 20% into predicted “Sensitive” and “Resistant” groups, respectively. Kaplan-Meier survival analysis revealed a significant separation in clinical outcomes between these cohorts (log-rank *P* = .020; HR =0.50, 95% CI: 0.28–0.91; see Fig. S7). We performed analysis of tumor-intrinsic embeddings encoded by the model (Fig. S8A). Specifically, we quantified the tumor-intrinsic pathway embedding coefficients across the predicted sensitive and resistant groups, identifying key pathways with significantly distinctive weight distributions between the two response cohorts. The sensitive group is characterized by high proliferative signatures, with significant enrichment in cell cycle-related pathways (e.g. ‘PRC2_EZH2’, ‘CYCLIN_D1’, and ‘RB_P107’), consistent with Cyclophosphamide’s mechanism of disrupting DNA during active replication. In contrast, resistance is associated with an escape from apoptosis characterized by TP53 surveillance (the primary checkpoint for DNA damage-induced death) loss and upregulated KRAS signaling. Similarly, we identified extrinsic factors distinguishing the two cohorts by analysing the TME-related embeddings. As shown in Fig. S8B, the sensitive group exhibited enrichment in the Complement pathway and markers of partial EMT. In breast cancer, partial EMT states often possess a higher proliferative advantage compared to fully mesenchymal or epithelial cells (Lüönd *et al.* 2021), thereby rendering them more vulnerable to alkylating agents. Conversely, the resistant group demonstrated a distinct metabolic shift toward Glycolysis and enriched Angiogenesis. These TME adaptations provide the bioenergetic precursors for cellular repair and a protective vascular niche, insulating resistant clones from therapeutic pressure (Peng *et al.* 2021).

## 4 Conclusion

In this study, we developed TMEDRP, a computational framework that shifts drug response prediction from a tumor-centric to a microenvironment-aware perspective. Unlike traditional methods that treat cross-domain variance as noise, TMEDRP distills these variations into interpretable representations through a pathway-informed disentanglement.

Experimental results confirm that integrating biological priors with uncertainty-driven adaptation significantly enhances model robustness. TMEDRP’s superior performance in cross-domain scenarios and independent clinical cohorts demonstrates its ability to capture generalizable pharmacogenomic patterns. Furthermore, BRCA case studies validate that the decoupled latent space effectively mirrors therapeutic mechanisms, proving that explicitly modeling the TME is necessary for translatable precision medicine.

Overall, TMEDRP provides a robust and interpretable tool for clinical drug response prediction, facilitating the translation of preclinical pharmacogenomic findings into clinical insights.

## Supplementary Material

btag680_Supplementary_Data

## Data Availability

The code and data used in this study are available on GitHub (https://github.com/kybinn/TMEDRP) and have been deposited in Zenodo (https://zenodo.org/records/22162842).
