# Supplementary material for: TMEDRP: decoding tumor-intrinsic and microenvironmental signatures for clinical drug response prediction
Source: Bioinformatics. 2026 Sep 12;42(9):btag680. doi: 10.1093/bioinformatics/btag680 (PMC13613059; doi:10.1093/bioinformatics/btag680)
Supplement: Supplementary file 1 [file btag680_supplementary_data.pdf]

Supplementary Materials for “TMEDRP: Decoding Tumor-Intrinsic and Microenvironmental Signatures for Clinical Drug Response Prediction”

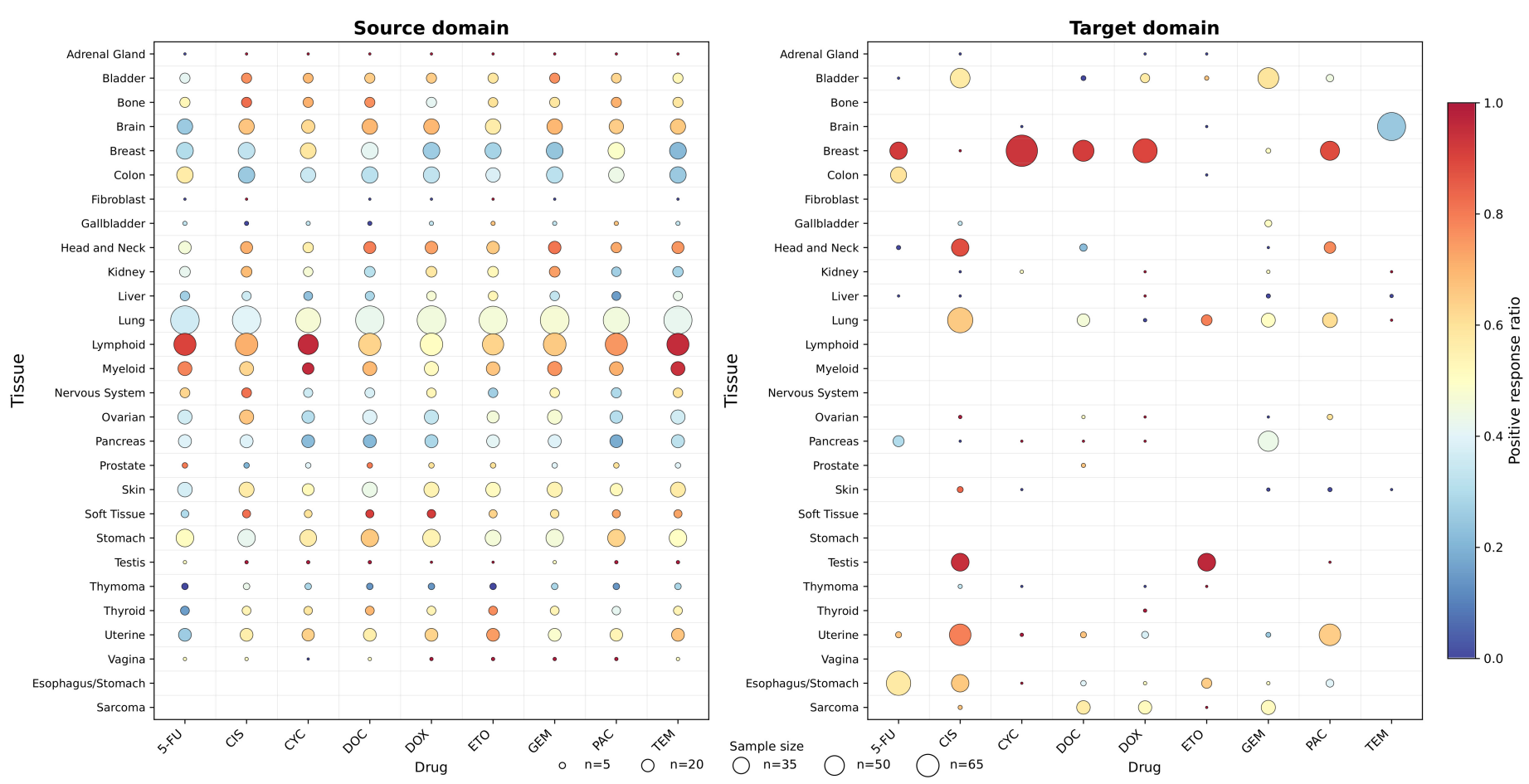

Figure S1: Drug-tissue sample distribution in the source and target domains. Bubble size denotes the total sample number, and bubble color denotes the proportion of drug-sensitive samples.

Table S1: Distribution of samples across GDSC, TCGA, and BLGSP datasets.

**Drug name**	**GDSC**		**TCGA**		**BLGSP (Unseen)**	
	**Samples**	**Pos. rate**	**Samples**	**Pos. rate**	**Samples**	**Pos. rate**
5-Fluorouracil	702	49.0%	220	59.0%	–	–
Cisplatin	702	55.4%	417	60.6%	–	–
Cyclophosphamide	578	56.1%	170	87.6%	91	73.6%
Docetaxel	701	51.0%	159	64.7%	–	–
Doxorubicin	707	49.0%	159	62.3%	62	83.9%
Etoposide	652	51.7%	96	64.5%	–	–
Gemcitabine	712	50.7%	230	39.0%	–	–
Paclitaxel	633	53.5%	222	60.8%	–	–
Temozolomide	694	57.8%	134	21.3%	–	–
Oxaliplatin*	–	–	38	52.6%	–	–
Epirubicin*	–	–	27	88.9%	–	–
Tamoxifen*	–	–	23	65.3%	–	–

Note: * unseen therapeutic agents in the test phase. "Pos. rate" denotes the proportion of positive (Sensitive) samples, calculated as Pos/(Pos + Neg).

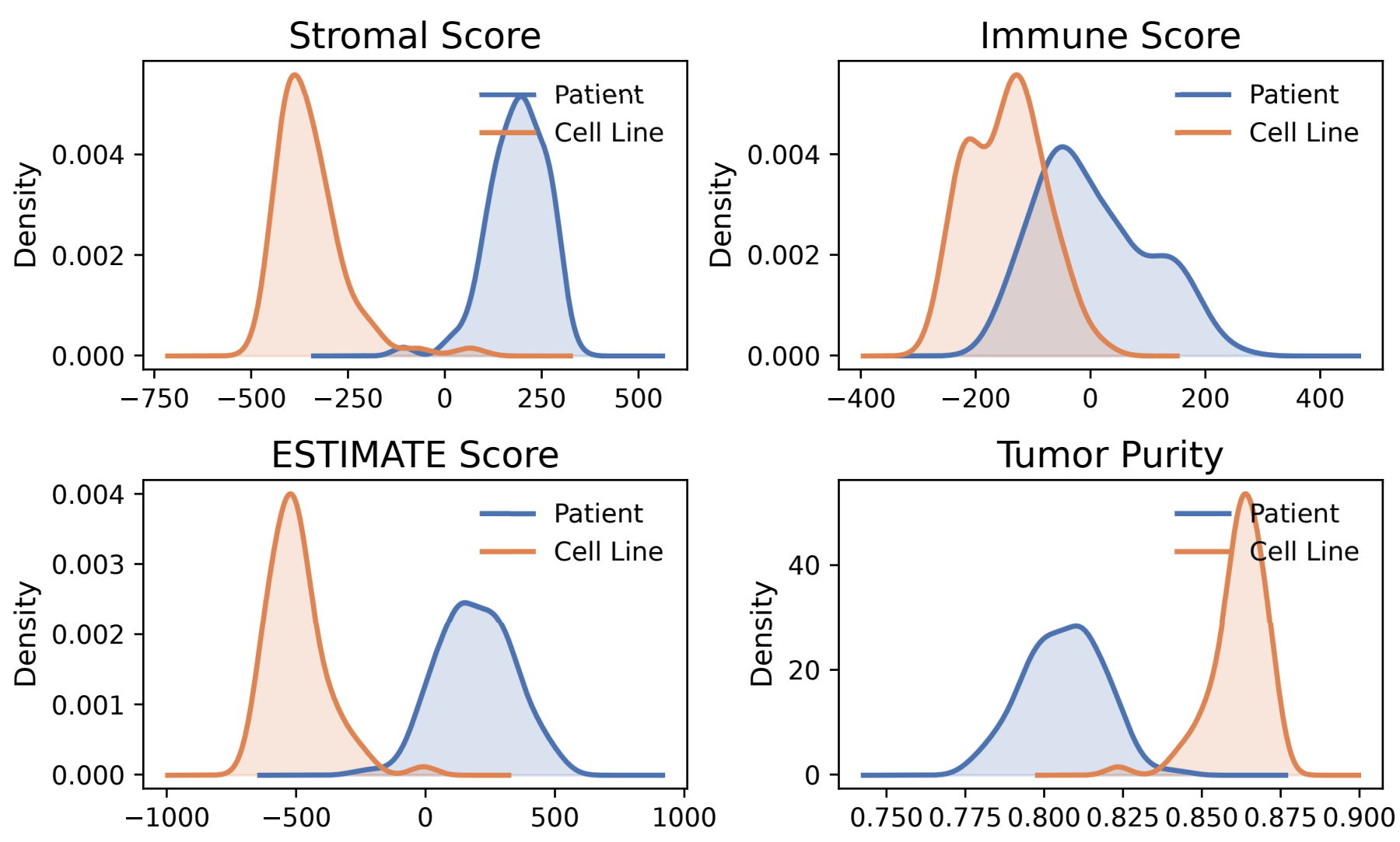

Figure S2: Distributional shifts in microenvironmental characterization between clinical and preclinical domains in Breast Cancer. Density plots compare the Stromal Score, Immune Score, and ESTIMATE Score alongside Tumor Purity for TCGA patient cohorts and CCLE cell lines.

Table S2: Tissue-specific sample distribution and positive response proportion in the source and target domains.

**Tissue type**	**GDSC**		**TCGA**	
	**Samples**	**Pos. rate**	**Samples**	**Pos. rate**
Adrenal Gland	9	88.9%	3	0.0%
Bladder	151	62.9%	165	55.8%
Bone	151	63.6%	–	–
Brain	335	60.9%	131	24.4%
Breast	405	34.3%	445	91.0%
Colon	373	35.4%	43	58.1%
Esophagus/Stomach	–	–	184	59.2%
Fibroblast	7	28.6%	–	–
Gallbladder	27	33.3%	11	45.5%
Head and Neck	212	68.9%	85	74.1%
Kidney	162	48.1%	7	57.1%
Liver	128	34.4%	8	12.5%
Lung	1135	43.3%	220	60.9%
Lymphoid	711	73.8%	–	–
Myeloid	277	72.9%	–	–
Nervous System	139	48.9%	–	–
Ovarian	275	41.1%	11	63.6%
Pancreas	249	31.3%	91	41.8%
Prostate	45	53.3%	3	66.7%
Sarcoma	–	–	96	54.2%
Skin	303	50.8%	13	38.5%
Soft Tissue	99	69.7%	–	–
Stomach	437	52.9%	–	–
Testis	16	87.5%	105	95.2%
Thymoma	63	19.0%	6	33.3%
Thyroid	115	53.0%	2	100.0%
Uterine	240	56.3%	178	69.1%
Vagina	17	70.6%	–	–

Note: "Pos. rate" denotes the proportion of positive (Sensitive) samples, calculated as Pos/(Pos + Neg).

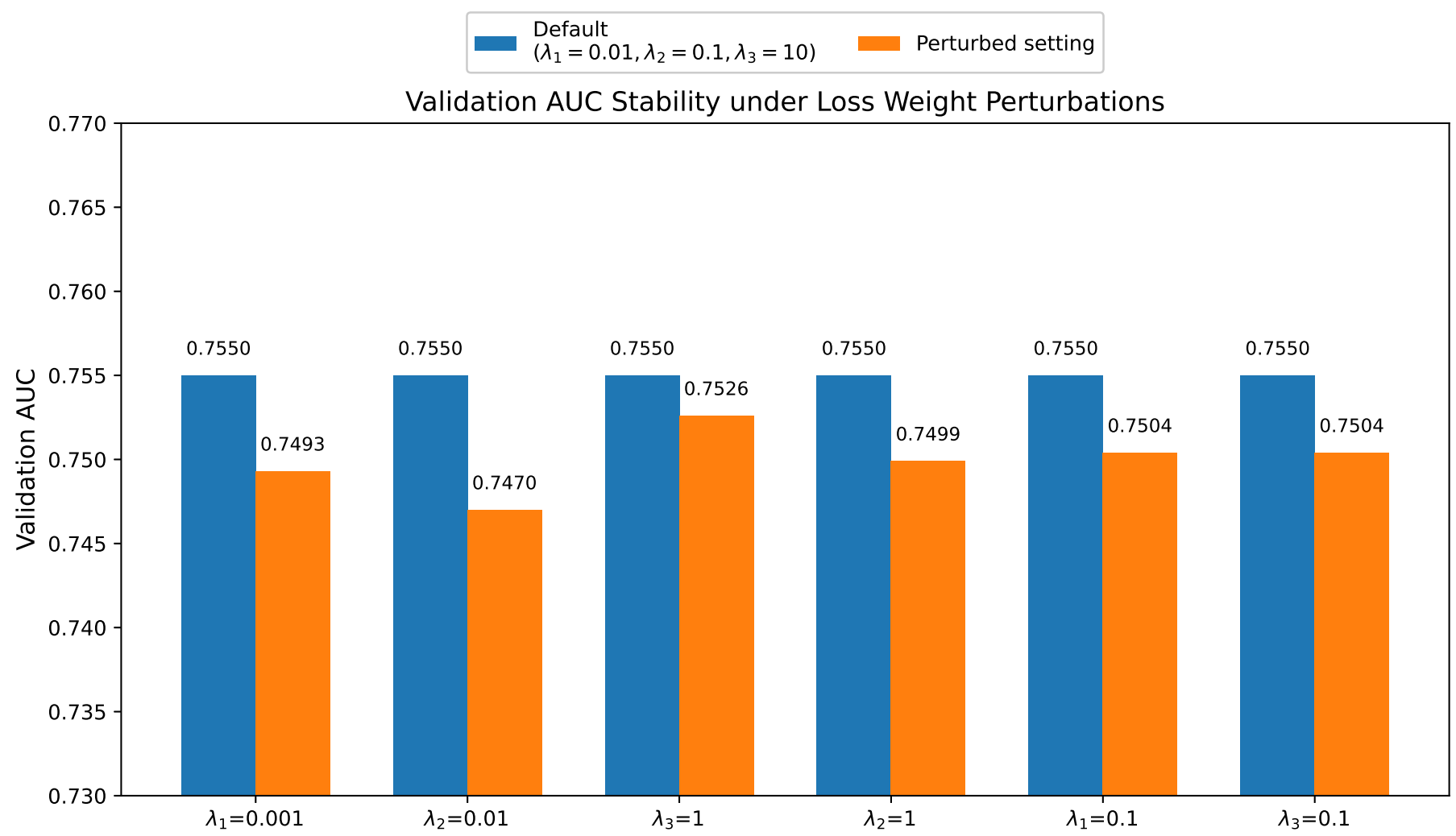

Figure S3: Sensitivity analysis of the loss weighting coefficients. Each coefficient was perturbed individually while the remaining coefficients were fixed at their default values $(\lambda_1, \lambda_2, \lambda_3) = (0.01, 0.1, 10)$. The validation AUROC ranged from 0.747 to 0.755 across the perturbations indicating that model performance was relatively stable with respect to these loss weights.

Table S3: AUROC performance comparison across nine therapeutic agents.

Method	5-FU	CIS	CYC	DOC	DOX	ETO	GEM	PAC	TEM	Mean
Velodrome	0.532	0.601	0.538	0.440	0.510	0.540	0.545	0.529	0.414	0.517
CODE-AE	0.558	0.601	0.385	0.448	0.376	0.743	0.561	0.481	0.289	0.494
WISER	0.549	0.592	0.496	0.469	0.454	0.637	0.555	0.513	0.294	0.506
PANCDR	0.540	0.611	0.522	0.476	0.553	0.622	0.536	0.486	0.317	0.518
drug2TME	0.505	0.521	0.507	0.529	0.423	0.507	0.504	0.491	0.478	0.496
TransDRP	0.532	0.629	0.526	0.367	0.369	0.753	**0.597**	0.493	0.650	0.546
DAPL	0.455	0.597	0.330	0.448	0.526	0.544	0.557	0.481	0.474	0.490
TRANSPIRE	0.559	**0.660**	0.524	0.494	0.478	0.609	0.552	**0.570**	0.417	0.540
MACB-DRP	0.476	0.599	0.589	0.379	0.360	**0.754**	0.565	0.442	0.667	0.537
**Ours**	**0.625**	0.637	**0.607**	**0.544**	**0.603**	0.725	0.586	0.529	**0.686**	**0.616**

Table S4: AUPR performance comparison across nine therapeutic agents.

Method	5-FU	CIS	CYC	DOC	DOX	ETO	GEM	PAC	TEM	Mean
Velodrome	0.639	0.769	0.918	0.656	0.754	0.845	0.516	0.702	0.227	0.670
CODE-AE	<u>0.683</u>	0.790	0.870	0.671	0.674	**0.933**	0.554	0.688	0.183	0.672
WISER	0.664	0.782	0.912	0.662	0.722	0.885	0.578	<u>0.717</u>	0.186	0.679
PANCDR	0.662	0.773	0.903	0.681	<u>0.771</u>	0.891	0.531	0.672	0.204	0.676
drug2TME	0.646	0.739	0.913	0.699	0.714	0.839	0.508	0.678	0.264	0.667
TransDRP	0.653	0.802	0.906	0.609	0.665	<u>0.928</u>	<u>0.585</u>	0.707	0.397	<u>0.695</u>
DAPL	0.607	0.783	0.865	0.633	0.746	0.854	0.545	0.676	0.261	0.663
TRANSPIRE	0.672	**0.823**	0.913	<u>0.708</u>	0.730	0.874	0.556	**0.732**	0.214	0.691
MACB-DRP	0.646	0.786	**0.930**	0.598	0.659	0.916	0.545	0.664	**0.502**	0.694
**Ours**	**0.727**	<u>0.814</u>	<u>0.928</u>	**0.716**	**0.794**	0.909	**0.601**	0.696	<u>0.466</u>	**0.739**

Table S5: F1-score results across nine therapeutic agents.

Method	5-FU	CIS	CYC	DOC	DOX	ETO	GEM	PAC	TEM	Mean
Velodrome	0.545	0.608	0.388	0.416	0.421	0.567	0.546	0.614	0.255	0.484
CODE-AE	0.583	0.674	0.422	0.401	0.307	0.845	0.568	0.532	<u>0.397</u>	0.525
WISER	0.028	**0.840**	0.763	<u>0.760</u>	0.332	0.546	0.494	0.490	0.324	0.509
PANCDR	<u>0.614</u>	0.585	0.787	0.760	<u>0.503</u>	<u>0.864</u>	<u>0.595</u>	<u>0.745</u>	0.355	<u>0.645</u>
drug2TME	0.499	0.568	0.595	0.562	0.494	0.598	0.467	0.529	0.297	0.512
TransDRP	0.317	0.551	0.175	0.320	0.283	0.806	0.468	0.327	0.033	0.364
DAPL	0.487	0.637	<u>0.810</u>	0.488	0.417	0.713	0.474	0.563	0.390	0.553
TRANSPIRE	0.347	0.395	0.234	0.279	0.114	0.246	0.177	0.473	0.117	0.265
MACB-DRP	0.552	0.740	0.793	0.326	0.279	0.857	0.511	0.468	0.240	0.529
**Ours**	**0.762**	<u>0.824</u>	**0.942**	**0.768**	**0.825**	**0.910**	**0.625**	**0.784**	**0.400**	**0.760**

Table S6: ACC Results across nine therapeutic agents.

Method	5-FU	CIS	CYC	DOC	DOX	ETO	GEM	PAC	TEM	Mean
Velodrome	<u>0.545</u>	0.608	0.388	0.416	0.421	0.567	0.546	0.614	0.255	0.484
CODE-AE	0.543	0.590	0.303	0.421	0.350	0.752	0.554	0.486	0.248	0.472
WISER	0.385	**0.724**	<u>0.747</u>	0.634	0.437	0.567	0.503	0.538	0.352	0.543
PANCDR	0.543	0.566	0.676	<u>0.643</u>	<u>0.514</u>	<u>0.773</u>	0.511	<u>0.637</u>	0.287	<u>0.572</u>
drug2TME	0.476	0.493	0.453	0.503	0.415	0.483	0.501	0.473	0.513	0.479
TransDRP	0.438	0.523	0.167	0.347	0.288	0.713	**0.584**	0.387	<u>0.746</u>	0.466
DAPL	0.441	0.561	0.687	0.438	0.419	0.625	0.543	0.495	0.360	0.508
TRANSPIRE	0.450	0.433	0.205	0.398	0.278	0.275	0.530	0.465	0.625	0.407
MACB-DRP	0.476	0.638	0.692	0.348	0.278	0.773	<u>0.556</u>	0.423	**0.749**	0.548
**Ours**	**0.636**	<u>0.708</u>	**0.893**	**0.649**	**0.727**	**0.840**	0.510	**0.657**	0.663	**0.698**

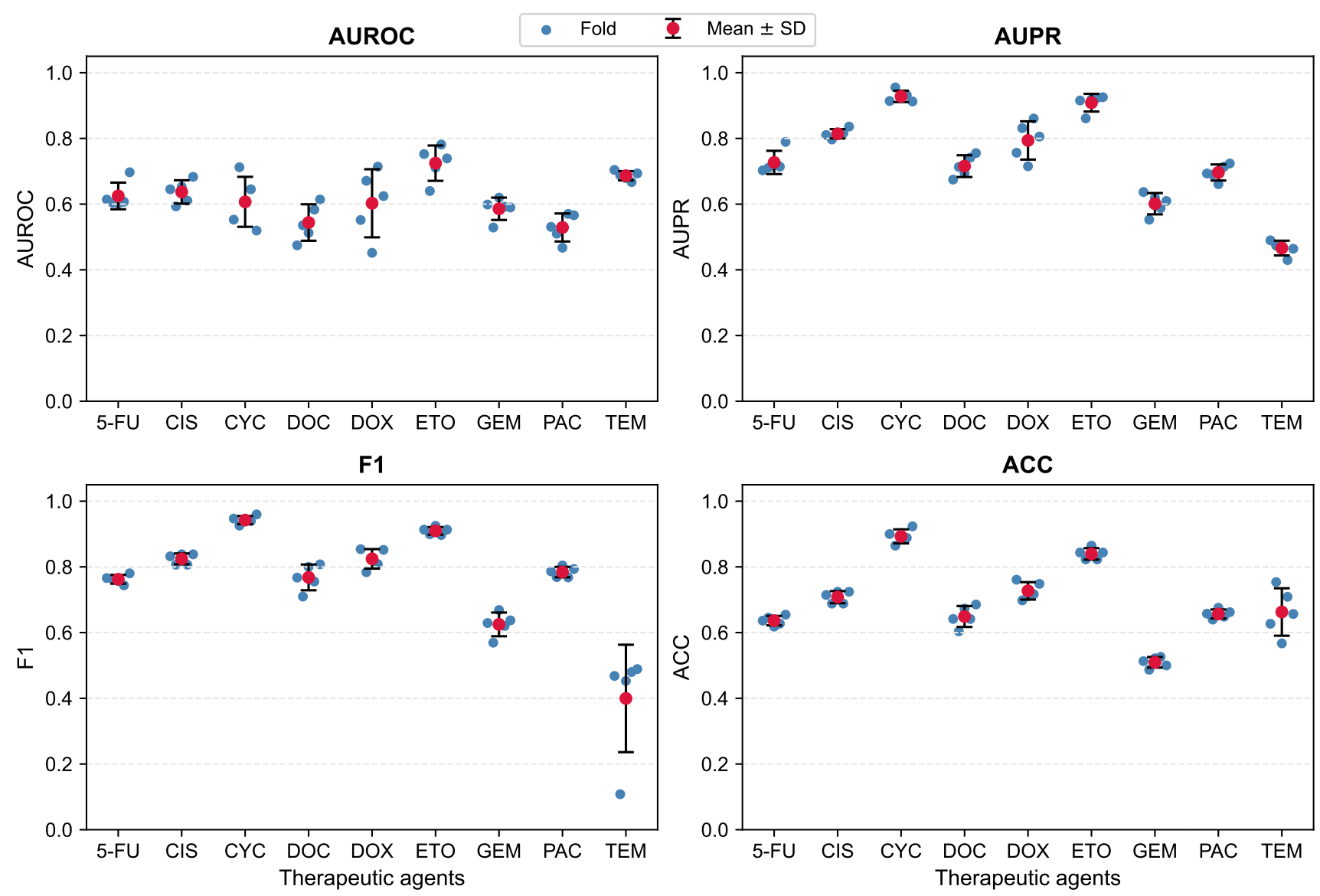

Figure S4: Five-fold variability of TMEDRP across nine therapeutic agents. Light-blue points represent the performance obtained in each individual fold, while red points and black error bars denote the mean and standard deviation (mean ± SD) across the five folds for AUROC, AUPR, F1-score, and ACC.

Table S7: Performance comparison on the BLGSP clinical cohort (Unseen Tissue).

**Method**	**CYC**		**DOX**	
	**AUC**	**AUPR**	**AUC**	**AUPR**
Velodrome	0.400	0.701	0.329	0.797
CODE-AE	0.418	0.718	0.467	0.814
drug2TME	<u>0.456</u>	0.727	0.458	0.808
PANCDR	0.409	0.696	0.367	0.791
WISER	0.444	<u>0.738</u>	0.317	0.748
DAPL	0.448	0.726	<u>0.483</u>	<u>0.855</u>
TRANSPIRE-DRP	0.366	0.653	0.464	0.793
**Ours**	**0.476**	**0.757**	**0.533**	**0.886**

Note: CYC: Cyclophosphamide; DOX: Doxorubicin. Bold: best; Underline: second-best.

Table S8: Performance comparison on unseen drugs.

Drug	Method	AUROC	AUPR
Oxaliplatin	drug2TME	0.503	0.598
	PANCDR	0.603	0.598
	DAPL	0.536	0.591
	**Ours**	**0.664**	**0.675**
Epirubicin	drug2TME	0.431	0.903
	PANCDR	0.444	0.910
	DAPL	0.289	0.834
	**Ours**	**0.583**	**0.930**
Tamoxifen	drug2TME	0.567	0.703
	PANCDR	0.367	0.562
	DAPL	0.283	0.578
	**Ours**	**0.617**	**0.739**

Note: Bold: best; Underline: second-best.

Table S9: Ablation study of TMEDRP across nine therapeutic agents (AUROC/AUPR).

Model	5-FU	CIS	CYC	DOC	DOX
Var. 1	0.517/0.645	0.598/0.768	0.538/0.911	0.393/0.630	0.419/0.695
Var. 2	0.526/0.648	0.562/0.752	0.462/0.903	0.471/0.683	0.441/0.718
Var. 3	0.612/0.724	0.608/0.779	0.564/0.927	0.433/0.644	0.516/0.761
Var. 4	0.613/0.708	**0.673**/0.810	0.598/**0.931**	0.424/0.652	0.430/0.708
Var. 5	0.614/0.709	0.648/0.792	**0.614**/0.925	0.414/0.647	0.477/0.730
**Full**	**0.625**/**0.727**	0.637/**0.814**	0.607/0.928	**0.544**/**0.716**	**0.603**/**0.794**

Model	ETO	GEM	PAC	TEM	Mean
Var. 1	0.554/0.841	0.539/0.531	0.447/0.646	0.560/0.341	0.507/0.645
Var. 2	0.519/0.823	0.541/0.533	0.484/0.675	0.408/0.255	0.490/0.631
Var. 3	0.638/0.858	0.570/0.571	0.509/**0.700**	0.661/0.422	0.568/0.710
Var. 4	0.701/0.877	0.570/0.560	0.468/0.680	**0.695**/**0.531**	0.575/0.718
Var. 5	0.702/0.891	0.581/0.563	0.493/0.681	0.605/0.389	0.583/0.692
**Full**	**0.725**/**0.909**	**0.586**/**0.601**	**0.529**/0.696	0.686/0.466	**0.616**/**0.739**

Note: Values are presented as AUROC/AUPR. Full model represents TMEDRP. Bold: best; Underline: second-best.

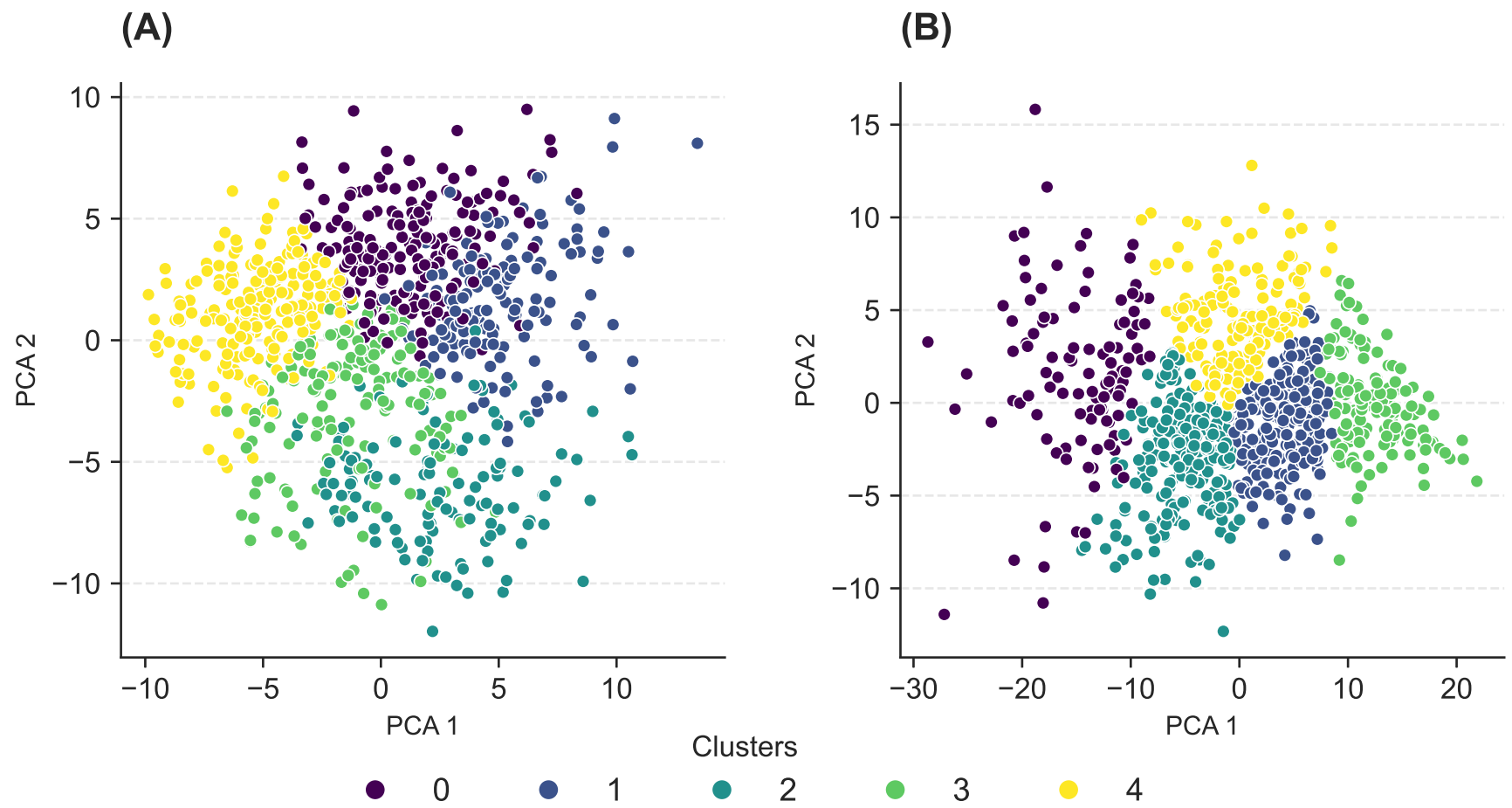

Figure S5: Visualization of Breast Cancer patient embeddings before (A) and after (B) the integration of the Neural Co-expression module. In alignment with clinical prior knowledge regarding the five major molecular subtypes of breast cancer, we pre-specified the number of clusters as five to evaluate the discriminative power of the patient embeddings.

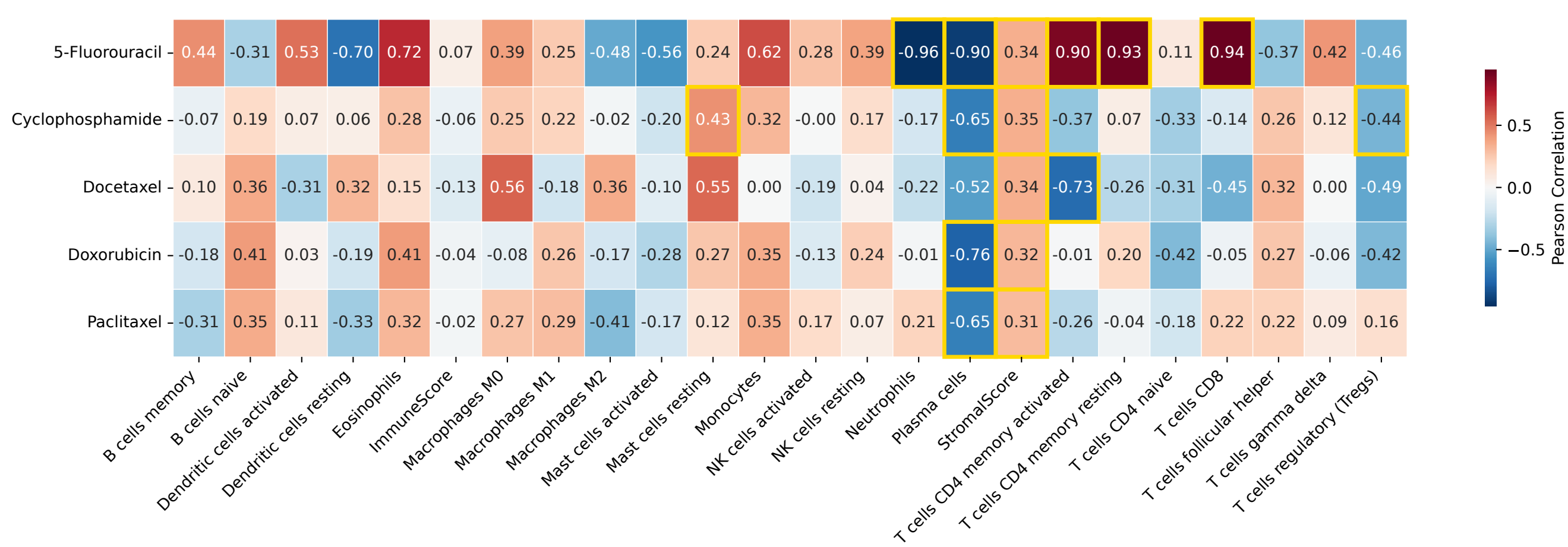

Figure S6: Pearson correlation between the first principal component (PC1) of the model-encoded TME representations and established microenvironment feature scores. PC1 explained 91.69%, 88.04%, 86.73%, 86.79%, and 86.54% of the total TME-embedding variance for 5-Fluorouracil, Cyclophosphamide, Docetaxel, Doxorubicin, and Paclitaxel, respectively. Significant correlations ($P < 0.05$) are highlighted in yellow.

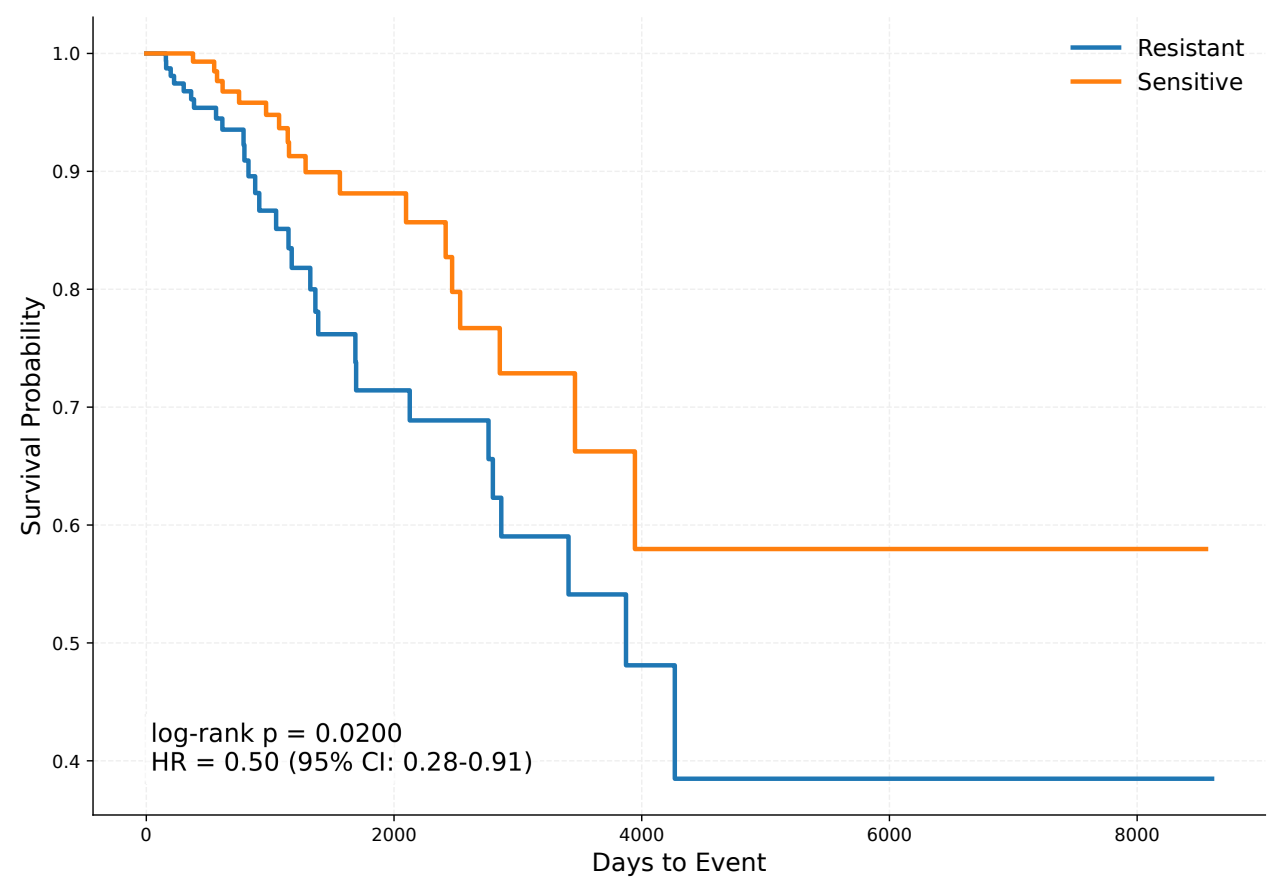

Figure S7: Kaplan-Meier survival analysis for predicted sensitive and resistant BRCA patients. Patients from the TCGA cohort were stratified by TMEDRP predicted scores for Cyclophosphamide (top and bottom 20%). The predicted groups showed significantly different survival outcomes (log-rank $p = 0.020$; HR = 0.50, 95% CI: 0.28-0.91).

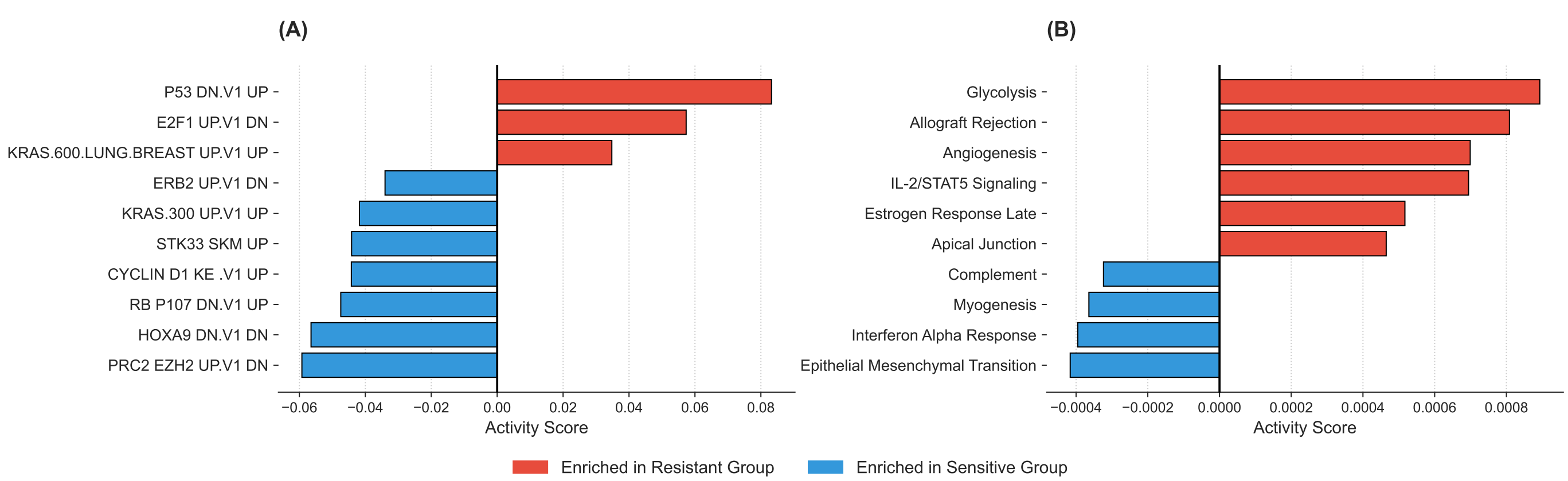

Figure S8: Differential pathway analysis. (A) Significantly differential distribution of cancer-intrinsic pathway scores derived from the cancer-related pathway encoder. (B) Significantly differential distribution of microenvironment-related pathway scores derived from the TME-related pathway encoder. Red bars indicate significant enrichment in the predicted resistant group, while blue bars represent pathways enriched in the sensitive group.

Table S10: TME-related pathways retained from the MSigDB Hallmark 2020 collection. The number of genes denotes the Genes overlapping with the 1,306-gene expression feature space. The 20 pathways contain 574 unique TME-associated genes in total. The detailed gene-to-pathway mapping relationships can be downloaded from our GitHub repository (`https://github.com/kybinn/TMEDRP`).

**No.**	**Hallmark pathway**	**Genes**
1	Epithelial Mesenchymal Transition	127
2	Estrogen Response Late	79
3	Hypoxia	77
4	TNF-alpha Signaling via NF-kB	72
5	Estrogen Response Early	67
6	Apical Junction	63
7	IL-2/STAT5 Signaling	58
8	Myogenesis	57
9	Coagulation	47
10	Glycolysis	56
11	Interferon Gamma Response	51
12	Complement	51
13	Inflammatory Response	45
14	Interferon Alpha Response	29
15	Angiogenesis	17
16	IL-6/JAK/STAT3 Signaling	26
17	Allograft Rejection	36
18	TGF-beta Signaling	17
19	Adipogenesis	34
20	Reactive Oxygen Species Pathway	11

Table S11: Oncogenic signatures retained from the MSigDB Oncogenic Signatures collection. The gene count denotes the number of TME-associated genes in each signature. Overall, 481 of 574 TME-associated genes are involved in at least one of the 85 retained signatures. Detailed mappings are available at `https://github.com/kybinn/TMEDRP`.

No.	Oncogenic signature	Genes	No.	Oncogenic signature	Genes	No.	Oncogenic signature	Genes
1	ESC V6.5 UP EARLY.V1 DN	55	30	ERB2 UP.V1 DN	24	58	AKT UP MTOR DN.V1 DN	15
2	EGFR UP.V1 UP	48	31	AKT UP MTOR DN.V1 UP	23	59	KRAS.50 UP.V1 UP	7
3	P53 DN.V1 UP	47	32	ATF2 UP.V1 DN	23	60	BCAT BILD ET AL UP	7
4	ESC J1 UP LATE.V1 UP	41	33	CYCLIN D1 KE.V1 UP	23	61	PRC2 EZH2 UP.V1 DN	15
5	BMI1 DN.V1 UP	35	34	MEL18 DN.V1 DN	20	62	GCNP SHH UP LATE.V1 DN	14
6	MEL18 DN.V1 UP	34	35	AKT UP.V1 DN	22	63	GCNP SHH UP EARLY.V1 DN	13
7	E2F1 UP.V1 DN	37	36	AKT UP.V1 UP	21	64	CYCLIN D1 KE.V1 DN	14
8	RAF UP.V1 DN	37	37	LEF1 UP.V1 DN	22	65	NFE2L2.V2	26
9	P53 DN.V1 DN	34	38	EGFR UP.V1 DN	22	66	RB P130 DN.V1 DN	11
10	MEK UP.V1 UP	34	39	PTEN DN.V2 UP	18	67	GCNP SHH UP LATE.V1 UP	13
11	STK33 NOMO UP	40	40	ESC J1 UP LATE.V1 DN	20	68	PTEN DN.V2 DN	11
12	KRAS.DF.V1 UP	32	41	MTOR UP.V1 UP	19	69	NOTCH DN.V1 DN	13
13	BMI1 DN MEL18 DN.V1 UP	28	42	BMI1 DN.V1 DN	17	70	WNT UP.V1 DN	12
14	RAF UP.V1 UP	31	43	SNF5 DN.V1 DN	18	71	PRC2 EDD UP.V1 UP	13
15	LEF1 UP.V1 UP	29	44	KRAS.600 UP.V1 UP	24	72	SIRNA EIF4GI DN	8
16	CAMP UP.V1 DN	29	45	KRAS.LUNG. BREAST UP.V1 UP	16	73	KRAS.300 UP.V1 DN	10
17	STK33 SKM UP	35	46	KRAS.300 UP.V1 UP	16	74	KRAS.KIDNEY UP.V1 UP	10
18	RB DN.V1 DN	23	47	KRAS.600.LUNG. BREAST UP.V1 UP	23	75	STK33 SKM DN	16
19	ESC J1 UP EARLY.V1 DN	27	48	ALK DN.V1 UP	15	76	KRAS.BREAST UP.V1 UP	10
20	RB P107 DN.V1 UP	24	49	ESC J1 UP EARLY.V1 UP	16	77	ATF2 S UP.V1 UP	12
21	MEK UP.V1 DN	28	50	MTOR UP.V1 DN	16	78	RB P107 DN.V1 DN	9
22	STK33 UP	34	51	SIRNA EIF4GI UP	11	79	BCAT GDS748 UP	5
23	ATF2 S UP.V1 DN	27	52	EIF4E DN	11	80	BCAT.100 UP.V1 UP	5
24	ESC V6.5 UP LATE.V1 UP	26	53	HOXA9 DN.V1 UP	16	81	WNT UP.V1 UP	11
25	SNF5 DN.V1 UP	25	54	RB DN.V1 UP	13	82	KRAS.600 UP.V1 DN	15
26	CYCLIN D1 UP.V1 UP	25	55	HOXA9 DN.V1 DN	16	83	KRAS.LUNG UP.V1 UP	9
27	BMI1 DN MEL18 DN.V1 DN	22	56	MTOR UP.N4.V1 UP	16	84	KRAS.DF.V1 DN	11
28	ESC V6.5 UP LATE.V1 DN	24	57	CAMP UP.V1 UP	16	85	P53 DN.V2 UP	9
29	ERB2 UP.V1 UP	24						
